# BAAT away liver cancer: conjugated bile acids impair T cell function in hepatocellular carcinoma immunotherapy

**DOI:** 10.1097/IN9.0000000000000062

**Published:** 2025-05-09

**Authors:** Zachary Detwiler, Snehal N. Chaudhari

**Affiliations:** 1 Wisconsin Institute for Discovery, University of Wisconsin-Madison, Madison, WI, USA; 2 Department of Biochemistry, University of Wisconsin-Madison, Madison, WI, USA

**Keywords:** conjugated bile acids, secondary bile acids, hepatocellular carcinoma, immunotherapy, T cells, CRISPR-Cas9

## Abstract

In this renaissance era of gene therapy, a new study published by the Susan Kaech lab in *Science* demonstrates the use of CRISPR-Cas9 technology to selectively deplete conjugated bile acids in the liver by targeting the bile acid–CoA:amino acid *N*-acyltransferase (*Baat*) gene to improve responsiveness to immunotherapy. This study highlights the role of conjugated bile acids in impairing intratumoral T cell function by directly accumulating in resident liver T cells and driving mitochondrial dysfunction. Knockout of *Baat* reduced hepatic conjugated bile acid production, thus improving immunotherapy potency and reducing tumor burden. Subsequently, *Baat* liver knockout reduced levels of microbially produced secondary bile acids such as lithocholic acid, a known carcinogen and T cell toxin. This study mechanistically links bile acids to liver cancer immunotherapy success, setting the stage for bile acid-based screening approaches and pharmacologic manipulations for improved patient outcomes.

Bile acids are multifaceted small molecules that play diverse physiological roles in the body. Primarily produced in the liver and actively reabsorbed by the intestine, the enterohepatic axis comprising the liver, gallbladder, intestine, and hepatic portal vein, harbors >95% of bile acid metabolites in the body ^[1]^. Therefore, bile acids play a major role in the gut-liver axis in health and disease. Several studies have investigated the role of individual bile acid metabolites on T cell function in the enterohepatic axis ^[2–5]^. In a tour de force study by Varanasi et al ^[6]^, a new role for conjugated bile acids has been discovered in influencing T cell function in hepatocellular carcinoma (HCC) immunotherapy. The authors find that conjugated bile acids and the expression of the bile acid–CoA:amino acid *N*-acyltransferase (*BAAT*) gene that produces conjugated bile acids is increased in human HCC tumors. CRISPR-Cas9 knockout of hepatic *Baat* significantly reduced conjugated bile acids and liver tumor burden in an HCC mouse model. Further, the authors show that in the absence of conjugated bile acids, there was an increase in infiltration of CD4^+^ and CD8^+^ T cells, natural killer (NK) cells, and an increase in pro-inflammatory immune responses in the liver, creating a conducive environment for liver cancer immunotherapy.

The authors find that conjugated bile acids accumulate in liver resident T cells and directly affect T cell survival. Taurine and glycine conjugated chenodeoxycholic acid (TCDCA and GCDCA) dose-dependently impacted T cell viability. Mechanistically, TCDCA impaired mitochondrial respiration and induced reactive oxygen species (ROS) production in T cells, suggesting oxidative stress as the primary cause of T cell death induced by conjugated bile acids. Interestingly, intratumoral CD8^+^ T cells showed minimal expression of bile acid exporters, suggesting that reduction of bile acid efflux by tumor-reactive T cells further potentiates T cell dysfunction and lack of immunotherapy responsiveness in HCC treatment. The authors further tested a panel of secondary bile acids on T cell function. Conjugated bile acids synthesized by the liver are further modified by the gut microbiome into unconjugated and secondary bile acids, predominantly lithocholic acid (LCA), deoxycholic acid (DCA), and ursodeoxycholic acid (UDCA). While LCA and DCA impaired T cell function, UDCA improved T cell health. Feeding HCC mouse models with LCA worsened tumor burden, while UDCA was very protective against HCC.

Previous studies have linked conjugated bile acids to anti-inflammatory and protective roles in the gut-liver axis ^[7–9]^. However, in the context of cancer immunotherapy, pro-inflammation can be advantageous to strengthen the immune response and enhance antitumor effects ^[10]^. Varanasi et al identified conjugated bile acids as one such restrainer of T cell responses, wherein the anti-inflammatory effects prove unfavorable in HCC immunotherapy. On the contrary, previous studies have found that pro-inflammatory effects of LCA and DCA on the liver further aggravate HCC progression ^[11,12]^. Together, these results suggest that in cancer immunotherapy, a delicate balance between pro- and anti-inflammatory responses must be maintained, as excessive inflammation can lead to immune-related adverse events ^[13]^. This study is thus revolutionary in identifying bile acids as a double-edged sword in the regulation of immune responses in HCC immunotherapy.

HCC is the most common liver cancer, but has a poor prognosis and limited treatment options, especially at late stages ^[14]^. Immune checkpoint blockade (ICB) therapy has been groundbreaking for HCC and works primarily by reinvigorating cytotoxic CD8^+^ T cells, which are repressed in the tumor microenvironment ^[15]^. However, the overall response rate in HCC for ICB is less than 30%, with common metabolic diseases further reducing response to therapy and increasing chances of reoccurrence ^[16]^. Varanasi et al suggest that *BAAT* expression or conjugated bile acid abundance in liver biopsies from HCC patients may allow prediction of response to immunotherapy. Fecal LCA or DCA levels, along with sequencing for microbiota commensals that produce these bile acids may also provide information on levels of T cell-impairing bile acids in enterohepatic circulation ^[17,18]^. Further, combinatorial therapies can be directed toward reducing bile acid accumulation in the HCC tumor microenvironment (Figure 1). Antisense oligonucleotide (ASO) and small interfering RNA (siRNA) therapies can be effectively and safely delivered to hepatocytes via lipid nanoparticles (LNPs) or via conjugation to *N*-acetylgalactosamine, as shown in FDA-approved drugs for metabolic diseases ^[19]^. These therapies could be used to transiently knock down *BAAT* during the course of ICB treatment to improve T cell function and cancer cell clearance. Chimeric antigen receptor (CAR) T cells targeting HCC are being developed and evaluated in clinical trials ^[20]^. CAR T and CAR NK cell therapies targeting solid tumors often utilize CRISPR-Cas9 editing to improve their function and persistence in the HCC tumor microenvironment ^[21]^. Knocking out bile acid importers in CAR T cells could be another strategy to improve their persistence and cytotoxic effects. Significant progress has been made in recent years in generating in vivo CAR T cells by delivering mRNA via LNPs ^[22]^. This same modality could also be used to deliver mRNA to increase the expression of bile acid exporters in T cells. Alternatively, siRNA could be delivered to knock down the expression of bile acid importers to tumor-fighting T cells to prevent bile acid accumulation. Developing small molecule inhibitors of hepatic bile acid conjugation or uptake proteins may increase the survival of cytotoxic T cells in the liver, further potentiating HCC clearance during ICB therapy.

**Figure 1.**
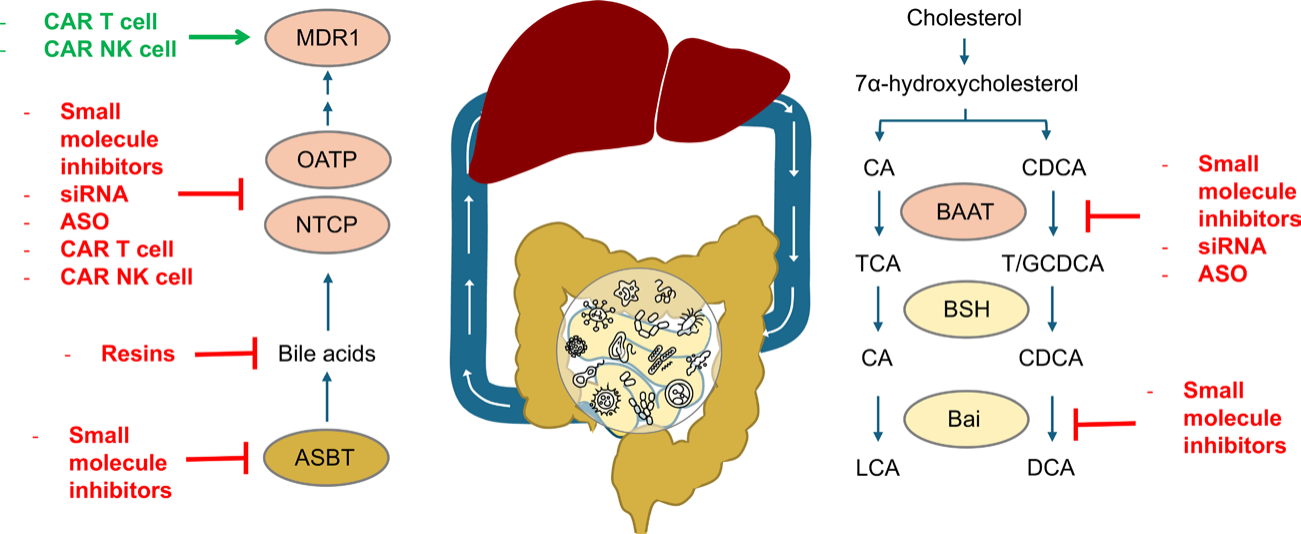
**Therapeutic strategies to reduce bile acid accumulation in hepatocellular carcinoma (HCC) tumor cells and microenvironment.** The liver synthesizes primary bile acids cholic acid (CA) and chenodeoxycholic acid (CDCA) sequentially from cholesterol. The bile acid–CoA:amino acid N-acyltransferase (BAAT) (right, pink) enzyme conjugates primary bile acids CA and CDCA to taurine (T) and glycine (G) conjugated bile acids. Conjugated bile acids are deconjugated by gut microbial bile salt hydrolase (BSH) enzymes and converted into secondary bile acids lithocholic acid (LCA) and deoxycholic acid (DCA) by the bile acid-inducible (Bai) operon (right, yellow). Intestinal bile acids are absorbed and recirculated predominantly by the apical sodium-dependent bile acid transporter (ASBT) protein (left, brown). Bile acids are imported into the liver by the importers organic anion transporter polypeptide (OATP) and sodium-taurocholate co-transporting polypeptide (NTCP) (left, pink). Bile acid efflux protein multidrug resistance 1 (MDR1) was shown by Varanasi et al to be the most dominant in mediating bile acid efflux in HCC (left, pink). Enzymes and transporters involved in increasing bile acid production and accumulation can be transiently inhibited by small molecule inhibitors. Alternatively, proteins can be transcriptionally silenced via antisense oligonucleotides (ASO) or small interfering RNA (siRNA). Engineering chimeric antigen receptor (CAR) T cell or CAR natural killer (NK) cells without bile acid importers or overexpressing bile acid exporters targeting the liver may induce HCC clearance. Targeting the microbiome BSH or Bai enzymes via small molecules may allow modulation of conjugated and secondary bile acids in HCC. Inhibition of bile acid reabsorption from the intestine or use of bile acid sequestrant resins may potentiate HCC immunotherapy by reducing bile acid levels in the tumor microenvironment.

Apart from targeting the liver directly, the Varanasi et al study reveals that modulating the gut microbiome or intestinal bile acid metabolism could serve as an adjunctive in HCC ICB therapy. Intestinal reabsorption of bile acids contributes significantly to the hepatic pool of conjugated and secondary bile acids ^[17]^. Inhibitors of bile acid transporters in the gut could also be used as a combinatorial therapeutic with ICB to maintain cytotoxic T cell function in the liver ^[23]^. Bile acid resins are FDA-approved intestinal sequestrants that block enterohepatic recirculation of bile acids and increase their excretion ^[24]^. Recent studies have found beneficial effects of bile acid sequestrants on digestive cancers ^[25]^. Finally, inhibitors of microbial LCA production or bile salt hydrolase activity would be beneficial in modulating levels of conjugated and secondary bile acids to test their role in HCC onset, progression, and treatments ^[18,26]^. Considering the role of the gut microbiome in modulating levels of bile acids in enterohepatic circulation, an investigation of how gut microbiome shifts may directly impact HCC therapy is warranted.

Conjugated bile acids circulate in the bloodstream to various other organs in the body ^[27]^. Whether these bile acids affect immunotherapy responses in other solid tumors is not yet known. Research in bile acid metabolism continues to advance, leading to discovery of new conjugated bile acid molecules made collaboratively by the host and the microbiome ^[28,29]^. Investigating the roles of newly discovered conjugated bile acids in potentiating immune responses in a variety of cancers may begin a new paradigm in immunometabolism research.

## Author contributions

Z.D. and S.N.C wrote, reviewed, and edited the manuscript.

## Conflicts of interests

Z.D. is a former employee and shareholder of CRISPR Therapeutics AG. S.N.C. is an ad hoc consultant for Metis Therapeutics.

## Funding

S.N.C. is supported by funding from the NIH (5R00DK128503-04), and Pfizer Inc.
